# Renal Accumulation and Hemocyte-Mediated Internalization After Acute Exposure to Injected Polyethylene Terephthalate Nanoplastics (PET-NPs) in the Freshwater Gastropod *Pomacea canaliculata*

**DOI:** 10.3390/jox16030088

**Published:** 2026-05-19

**Authors:** Anita Ferri, Sandro Sacchi, Chiara Losi, Martina Amico, Nicola Franchi, Davide Malagoli

**Affiliations:** 1 Department of Life Sciences, University of Modena and Reggio Emilia, Via Giuseppe Campi 213/D, 41125 Modena, Italy; anita.ferri@unimore.it (A.F.); sandro.sacchi@unimore.it (S.S.); nicola.franchi@unimore.it (N.F.); 2 Department of Chemical and Geological Sciences, University of Modena and Reggio Emilia, Via G. Campi 103, 41125 Modena, Italy; chiara.losi@unimore.it; 3 Independent Researcher, 41125 Modena, Italy; 4 NBFC—National Biodiversity Future Center, 90133 Palermo, Italy

**Keywords:** apple snail, environmental immunology, immune-response, invertebrate immunocyte, mollusk, phagocytosis, plastic, pollution, snail kidney

## Abstract

The increasing fragmentation of plastic debris into nanosized particles represents a threat to freshwater ecosystems, yet the biological effects of nanoplastics (NPs) on freshwater invertebrates remain poorly understood. This study investigated tissue distribution, cellular effects and immune responses following acute exposure to polyethylene terephthalate nanoplastics (PET-NPs) in the freshwater gastropod *Pomacea canaliculata*, a species of high ecological relevance and physiological resilience. Adult snails were injected with PET-NPs at 5 or 10 mg/L and sampled after 24 and 72 h. PET-NPs accumulation in the anterior and posterior kidneys was assessed by fluorescence imaging and tissue morphology was evaluated. Stress- and inflammation-related genes (Pc-Heat Shock Protein (HSP)70, Pc-HSP90 and Pc-Allograft inflammatory factor 1) expression was quantified by RT-qPCR. PET-NPs uptake and phagocytic activity were analyzed in circulating hemocytes in vivo and ex vivo. PET-NPs were accumulated in renal tissues, persisting up to 72 h without histopathological alterations. Gene expression analyses revealed non-linear and dose/time-dependent responses. Hemocytes of different morphologies internalized PET-NPs in a dose-dependent manner and showed intercellular particle transfer. Overall, acute PET-NP exposure determines rapid immune handling and tissue sequestration with limited short-term physiological impact, underscoring the potential involvement of immune processes in NPs fate and highlighting the need for chronic exposure studies.

## 1. Introduction

Plastic has become an integral part of modern society, and since the mid-20th century, global plastic production has increased exponentially, reaching an estimated 413 million tons in 2023 [1,2,3]. Research indicates that between 22% and 43% of mismanaged plastic waste accumulates in landfills or is transported by rivers to the oceans, thereby contributing to the global dispersal of persistent plastic debris [4,5]. Plastic products are characterized by their extreme resistance to degradation, which occurs under physical, chemical or biological stress, leading to persistence over prolonged ecological timescales and contamination of different environments, including remote polar regions, deep-sea sediments, and high-altitude ecosystems [6,7,8,9,10,11].

As larger plastic items progressively fragment, they generate microplastics (MPs, particles < 5 mm) and NPs (NPs; particles < 100–1000 nm, depending on the classification adopted) [12,13]. Although early research predominantly focused on MPs, there is now a growing interest in nanoscale particles due to their enhanced reactivity, bioavailability and ability to interact directly with cellular structures [12]. NPs may be produced intentionally for industrial applications, or they may arise secondarily from environmental and unregulated degradation processes [14,15,16].

Due to their extremely small size, NPs can cross biological barriers that are typically impermeable to larger microplastic fragments [17]. While MPs tend to induce mechanical stress (gut blockage, reduced feeding, abrasion), NPs exert more profound physiological toxicity because of their ability to penetrate tissues and alter intracellular homeostasis even at low concentrations [17]. They can be incorporated not only through the digestive system but also across gills, epithelia, or even directly through cellular membranes [13]. Once internalized, NPs can reach organelles, interfere with signaling pathways, disrupt mitochondrial function, and generate reactive oxygen species, leading to oxidative stress, inflammation, apoptosis, or metabolic dysregulation. These effects have been documented in diverse taxa, including mammals, fish, crustaceans, bivalves, and other aquatic terrestrial invertebrates, and frequently involve impairments in immunity, growth, reproduction, and behavior [17]. Such observations underscore the importance of investigating the biological impact of NPs on ecologically relevant species, especially those likely to encounter these contaminants due to their habitat or feeding strategy.

Of all the different types of polymer, polyethylene terephthalate (PET) has become particularly ubiquitous. PET is a linear thermoplastic polymer composed of ethylene glycol and terephthalic acid monomers, widely used in films, fibers, textiles, and beverage packaging, thanks to its mechanical resistance and high melting point [18]. Global demand for amorphous PET is especially high in Asia, primarily driven by bottle manufacturing [4]. However, PET has a low recycling efficiency, with only 42% of recycled bottles, while the rest are incinerated or laid to accumulate in landfills, where they break down into MPs and NPs over time [4]. Studies on experimental organisms demonstrate that PET-NPs can induce oxidative stress, nuclear damage, metabolic alteration, and reduced survival, as observed, for example, in *Drosophila melanogaster*, highlighting the cellular and systemic toxicity of PET particles and their potential ecological relevance [19].

Despite growing awareness of PET particle contamination, there are still significant gaps in our understanding of the occurrence, distribution, and ecological impact of PET-NPs, particularly in freshwater ecosystems. This is problematic because freshwater bodies, such as rivers, streams, lakes and irrigation channels, are in close proximity to human settlements, agricultural runoff, wastewater discharge sites and industrial land-based pollution sources. These systems often receive high concentrations of plastic and associated contaminants, and their sediments may accumulate concentrations that are even higher than those typically found in marine environments [20]. Freshwater organisms are therefore directly and chronically exposed to complex mixtures of MP/NPs particles, and the lack of data is a critical limitation for environmental risk assessment [21]. Existing research on plastics in freshwater is mainly focused on fish and small crustaceans, with fish accounting for over half of all studies and cladocerans, such as *Daphnia magna*, representing a significant proportion of invertebrate models [20,22]. However, ecotoxicological and ecoimmunological investigations addressing the mechanistic and immunological effects of PET-NPs in freshwater species remain scarce, and the identity of the most sensitive endpoints remains unclear.

In this context, *Pomacea canaliculata* is a valuable freshwater gastropod model for NPs research. Originally from South America, this species is now considered as one of the world most invasive mollusks, occupying a wide range of freshwater habitats including streams, lakes, irrigation canals and wetlands, where temperatures range between 18 and 25 °C but populations can persist in conditions ranging from 10 to 35 °C due to their notable thermal tolerance [23,24]. *P. canaliculata’s* ecological success is supported by high physiological plasticity, resistance to drought, and robust responses to environmental stressors [25]. *P. canaliculata* specimens can thrive in heavily contaminated waters, their gills can accumulate high concentrations of heavy metals with minimal mortality, and histopathological changes in the digestive tract occur without compromising survival [26]. Furthermore, *P. canaliculata* has a functional neuro-immune system and shows innate immune memory after a bacterial challenge, making it a promising organism for studying invertebrate immunity [27,28,29].

Due to their small size, micro- and nanoplastics (MPs/NPs) can easily interact with the immune system, particularly innate immune cells and molecules, and can interfere with signaling pathways essential for cellular homeostasis [30]. Innate immunity is the body’s primary defense against foreign materials, including MPs/NPs and is mediated by evolutionarily conserved effector cells that act through processes such as phagocytosis, the production of reactive oxygen and nitrogen species, and the release of lysosomal enzymes, antimicrobial peptides, and inflammatory mediators [31]. Interactions between NPs and innate immunity have been documented across a wide range of taxa, including invertebrates such as mollusks, arthropods, and echinoderms, as well as vertebrates [30,32].

Species-specific differences in immune sensitivity to NP exposure have been reported. For example, *Ciona robusta* appears to be impaired in its immune response to environmental stressors when exposed to NPs [33], whereas other organisms display more robust responses [34].

At the cellular level, NPs can be internalized by phagocytes and trafficked to lysosomal compartments, where they can induce oxidative stress, apoptosis and functional impairments [35]. A growing body of evidence indicates that the physicochemical properties of NPs can significantly impact these processes, resulting in altered immune functions, increased susceptibility to pathogens and disrupted immune signaling pathways. Distinct responses have been observed among different hemocyte subpopulations, with granular hemocytes, which correspond to fully mature phagocytes, being preferentially involved [36].

In addition to cellular defenses, soluble factors present in the hemolymph contribute to innate immunity by interacting with non-self-materials, typically through the recognition of conserved structural motifs rather than specific ligands [37]. Studies employing surface-modified nanoparticles (NPs) have further highlighted the pivotal role of individual hemolymph proteins in particle recognition and immune modulation [38].

Despite living in environments where MP/NPs contamination is likely to be significant [39,40,41,42], and despite its physiological attributes making it highly suitable for research focused on environmental impact on immune functions, to our knowledge, no studies have investigated the impact of PET-NPs on *P. canaliculata*. Unlike other freshwater organisms, dominating ecotoxicology studies, *P. canaliculata* combines key traits rarely found in a single model organism: tolerance to contaminated environments, accumulation of pollutants in target organs, and a well-studied innate immune system. This makes it uniquely suited to investigate both the cellular and systemic immunotoxicological effects of PET-NPs.

In this study, we examined the effects of acute exposure (24–72 h) to PET-NPs delivered by feeding first and injection secondly at concentrations of 5 and 10 mg/L. We assessed the accumulation of fluorescent PET-NP in circulating immunocyte and immune-related and hemocyte-containing organs, using cryosectioning and software driven image analysis. Given the absence of accumulation through feeding exposure, we performed further analysis only on animals subjected to intraperitoneal exposure. Histological alterations following PET-NPs accumulation and the expression of immune- and stress-related molecules (HSP70, HSP90 and AIF1) were assessed as read-outs for NP effects. Furthermore, the phagocytic activity of circulating hemocytes towards PET-NPs was evaluated both in vivo and in vitro to determine whether immune cells can internalize PET-NPs. To our knowledge, this is the first study to investigate the immunotoxicological effects of PET-NPs in *P. canaliculata*, addressing a critical gap in our understanding of PET-NPs impacts on freshwater gastropods. By combining in vivo and in vitro approaches, fluorescence-based particle tracking, histological analysis, and molecular markers of stress and immune activation, this study provides a multi-level characterization of PET-NP effects on innate immunity that has not previously been attempted in this ecoimmunologically relevant species.

## 2. Materials and Methods

### 2.1. Animal Husbandry and Acute Exposure to PET-NPs

Animals were maintained under laboratory conditions as previously described in Ferri et al., 2025 [29] in the authorized aquatic facility of the Department of Life Sciences, University of Modena and Reggio Emilia, continuously since 2008. Individuals were kept in dechlorinated tap water at 25 ± 1 °C under a 12 h light/dark photoperiod. Water quality was maintained by replacing approximately 90% of the tank volume twice weekly, after which animals were fed a mixture of leafy vegetables commonly used in the human diet. Specimens approximately 18 months old were used for the experiments. Prior to exposure, animals were anesthetized and injected into the foot with Polyethylene terephthalate nanoplastics (PET-NPs) suspensions prepared in filtered Freshwater Snail Solution (FSS: 41.15 mM NaCl, 0.54 mM KCl, 3.55 mM CaCl2, 2.61 mM MgCl2, 5 mM Tris, pH 7.5 [43]). PET-NPs were provided by Dr. Davide Tessaro (Politecnico of Milan, Milan, Italy) and consisted of fluorescently labeled particles (Nile Red; excitation 559 nm, emission 635 nm) with a mean diameter of 82 nm [44].

Prior to the injection experiment, a preliminary dietary exposure trial was conducted to assess whether oral administration of PET-NPs could serve as a feasible exposure route. Three animals were fed PET-NP-spiked gelatin pellets (1% agar, 5 mg/L PET-NPs in freshwater, 1 mL per pellet) once daily for five consecutive days, followed by a two-day fasting period; three additional animals received unspiked pellets and served as controls. However, fluorescence microscopy analysis of target organs revealed no detectable accumulation of PET-NPs following dietary exposure (Supplementary Figure S5), suggesting that oral uptake was insufficient to deliver measurable particle loads to tissues under the conditions tested.

Immersion exposure was also considered but deemed unsuitable for several technical reasons: it would have required impractically large volumes of water and PET-NP suspensions, risked contamination of rearing tanks, and would not have allowed reliable control of the actual dose absorbed by each animal, particularly given the tendency of *P. canaliculata* to retract and seal the shell with the operculum when disturbed. Injection was therefore selected as the exposure route, as it ensures controlled and reproducible delivery of a defined PET-NP dose directly into the hemolymph. This parenteral approach has been previously employed in our laboratory for exposure to other types of nanoparticles, providing a validated framework for the present investigation [45].

A total of six experimental groups were established (5 animals per group):

(i) control animals injected with FSS+ HFIP (hexafluor-2-propanol) 0.0035% solution (PET-NPs vehicle) [44] and sacrificed 24 h post-injection (hpi);
(ii) control animals injected with FSS + HFIP 0.0035% and sacrificed 72 hpi;
(iii) animals injected with 5 mg/L PET-NPs and sacrificed 24 hpi;
(iv) animals injected with 5 mg/L PET-NPs and sacrificed 72 hpi;
(v) animals injected with 10 mg/L PET-NPs and sacrificed 24 hpi;
(vi) animals injected with 10 mg/L PET-NPs and sacrificed 72 hpi.

For each injection [46], a total volume of 500 µL was administered. From injection until sacrifice, animals were maintained in tanks identical to those used for rearing, but were kept under fasting conditions.

### 2.2. Hemolymph Collection and Dissection of Target Organs

For PET-NP-treated groups, hemolymph withdrawal was performed prior to organ dissection. Hemolymph was collected by applying gentle and continuous pressure at the level of the operculum. In response, animals retracted into the shell and released hemolymph, which was collected into tubes and kept on ice until further processing. Following hemolymph collection, animals were anesthetized on ice for 30 min to allow dissection of the target organs, namely the anterior kidney (AK) and posterior kidney (PK). For each organ, tissue samples were subdivided and processed for cryosectioning, histological analysis and RNA extraction.

### 2.3. PET-NPs Accumulation in Target Organs and Hemocytes

Dissected tissues were fixed by immersion in isopentane (Sigma, St. Louis, MO, USA) under liquid nitrogen vapor for 1 min. Fixed AK and PK samples were then oriented on suitable supports, embedded in Optimal Cutting Temperature (OCT) compound, and stored at −20 °C until sectioning. Samples were sectioned at a thickness of 20 µm using a cryostat (Leica CM 1510 S), and sections were mounted on Superfrost™ Plus adhesion microscope slides (Epredia Italy, Milan, Italy). Slides were mounted with an aqueous mounting medium (Bio-Optica, Milan, Italy) and observed using a Nikon Eclipse microscope (Nikon Europe B.V, Amstelveen, The Netherlands).

### 2.4. Morphological Analysis of AK and PK

The collected organs were fixed in freshly prepared Bouin’s solution. The fixed tissue samples were washed in 70% ethanol and progressively dehydrated using graded ethanol solutions (70%, 95%, and 100%), subsequently cleared in xylene, and embedded in Paraplast^®^ (Sigma-Aldrich, Merck, Darmstadt, Germany). Paraffin blocks were sectioned at a thickness of 7 µm with a rotary microtome, and the sections were deposited on StarFrost^®^ microscope slides (Sigma-Aldrich, Merck). Before histological processing, sections were subjected to routine deparaffinization and rehydration through descending ethanol concentrations (100%, 95%, and 70%), followed by xylene treatment, according to established protocols [47]. Prepared slides were then stained with Mayer’s hematoxylin and eosin (H&E). Slides were mounted with Eukitt ^®^ (Sigma-Aldrich, Merck) mounting media and observed using a Nikon Eclipse microscope (Nikon Europe B. V., Amstelveen, The Netherlands).

### 2.5. Stress and Inflammation Related Gene Expression Analysis

Total RNA was isolated using the ReliaPrep™ RNA Miniprep System (Promega, Madison, WI, USA) according to the manufacturer’s guidelines. RNA concentration and purity were assessed spectrophotometrically using a NanoDrop™ ND1000 (Thermo Fisher Scientific, Waltham, MA, USA). For each sample, approximately 1 µg of total RNA was reverse-transcribed into complementary DNA (cDNA) using the iScript cDNA Synthesis Kit (Bio-Rad Laboratories, Inc., Hercules, CA, USA), following the supplied protocol. The resulting cDNA was used as a template for quantitative real-time PCR (qPCR) analyses performed with the SsoAdvanced™ Universal SYBR^®^ Green Supermix (Bio-Rad Laboratories, Inc.). Gene-specific primers were used to amplify the reference gene 60S ribosomal protein L5-like (Pc-RpL5) and the target genes Pc-HSP70, Pc-HSP90, and Pc-AIF1 (Table 1). Amplifications were carried out according to the manufacturer’s instructions. The qPCR cycling program consisted of an initial denaturation step at 95 °C for 2 min, followed by 30 amplification cycles at 95 °C for 10 s and 60 °C for 30 s. All reactions were run in technical triplicate using a CFX-Duet Real-Time PCR Detection System (Bio-Rad Laboratories, Inc.) at the National Biodiversity Future Center (NBFC), University of Modena and Reggio Emilia (Modena, Italy). At the end of each run, melting curve analysis was performed to confirm amplification specificity.

Relative gene expression levels were calculated using the 2^−ΔΔCt^ method [48]. Data are reported as fold changes relative to the control group, which was set to 1. Expression values were normalized against Pc-RpL5. Prior to statistical analysis, data distribution and variance homogeneity were verified using the Shapiro–Wilk test and the F-test, respectively (*p* > 0.05). Differences among groups were evaluated by one-way ANOVA followed by Tukey’s post hoc test or Kruskall–Wallis followed by Mann–Whitney U test using PAST software [49]. Statistical significance was set at α = 0.05.

### 2.6. Immunohistochemical Experiments on Circulating Hemocytes from Treated Snails

Hemocytes collected from treated animals were used to prepare microscope slides for nuclear staining with DAPI or for immunofluorescence analysis of the actin cytoskeleton, followed by DAPI counterstaining. For slide preparation, freshly collected hemolymph was gently deposited onto glass slides and allowed to adhere for 10 min at room temperature. Excess liquid was then carefully removed and cells were fixed for 10 min using 4% paraformaldehyde prepared in 1× phosphate-buffered saline (4% PFA in PBS).

For nuclear staining, hemocytes were incubated with DAPI solution (final concentration 1 µg/mL) for 10 min in the dark. After removal of the staining solution, the slides were mounted with an aqueous mounting medium. These preparations were used to assess the presence and intracellular localization of PET-NPs within circulating hemocytes.

Immunofluorescence labeling of actin was performed to better evaluate the morphology of hemocytes involved in PET-NP phagocytosis. Cells adhered to slides were fixed for 10 min using 4% PFA in PBS. Following fixation, slides were treated with a methanol/hydrogen peroxide solution to quench endogenous peroxidase activity and reduce nonspecific background signals. Slides were subsequently rinsed in ultrapure water and washed three times in 1× PBS (pH 7.4).

To block nonspecific antibody binding, slides were incubated for 30 min with a blocking solution consisting of 1× PBS supplemented with 2% normal goat serum (DBA Italia, Segrate, Italy). Hemocytes were then incubated overnight at 4 °C with the primary anti- actin antibody (MABT826 Anti-Actin Antibody, clone 2G2, Sigma-Aldrich, Merck) diluted 1:500 in blocking solution. After three washes in 1× PBS, slides were incubated for 2 h at room temperature with a fluorescently labeled secondary antibody (Goat anti-Mouse IgG (H+L) Highly Cross-Adsorbed Secondary Antibody, Alexa Fluor™ Plus 488 A32723, ThermoFisher Scientific, Invitrogen, Carlsbad, CA, USA) diluted 1:1000 in blocking solution, followed by additional 1xPBS washes. Nuclei were counterstained with DAPI (Sigma-Aldrich, Merck) as described above, excess stain was removed, and slides were mounted using Mowiol^®^ (Sigma-Aldrich, Merck). Negative controls lack the primary antibody.

All slides were examined using a Nikon Eclipse fluorescence microscope. Images were acquired using specific excitation wavelengths for DAPI to visualize nuclei (Ex peak 359 nm), FITC to detect the actin cytoskeleton (Ex peak 495 nm), and Texas Red to detect PET-NPs (Ex peak 586 nm). Fluorescence channels were acquired separately and successively overlaid to assess intracellular accumulation of PET-NPs and hemocyte morphology.

### 2.7. Ex Vivo Hemocyte Exposure to PET-NP

Hemocytes were collected from control animals as described above and used for ex vivo exposure experiments to quantify PET-NP uptake. Aliquots of hemolymph were exposed to PET-NPs in 2 mL plastic tubes at a final concentration of 5 mg/L or 10 mg/L. As *P. canaliculata* hemocytes quickly clot after withdrawal, a subset of hemolymph samples was supplemented with sodium citrate (10 mM final concentration). Parallel samples were diluted in an equivalent volume of filtered hemolymph to ensure identical cell dilution across treatments. In all cases, 500 µL of hemolymph containing hemocytes were mixed at a 1:1 ratio with either filtered hemolymph or sodium citrate solution prior to PET-NP addition.

Hemocyte suspensions were incubated for 30 min under gentle agitation to allow interaction with PET-NPs. Following incubation, samples were deposited onto glass slides and allowed to adhere for 10 min at room temperature. Then, the cells were fixed, counterstained with DAPI, and mounted with Mowiol^®^ (Sigma-Aldrich, Merck).

Each treatment was performed in triplicate, with three slides prepared per experimental condition. Slides were examined using a Nikon Eclipse fluorescence microscope. Images were acquired using excitation wavelengths specific for DAPI to visualize nuclei (Ex peak 359 nm) and Texas Red to detect PET-NPs (Ex peak 586 nm). Fluorescence channels were acquired separately and successively overlaid to evaluate intracellular PET-NP accumulation within hemocytes.

### 2.8. Image Acquisition and Analysis

Cryostat sections were imaged under both bright-field DIC and fluorescence microscopy using a red fluorescence channel (excitation peak at 586 nm). Corresponding bright-field and fluorescence images were overlaid for analysis. Quantification of PET-NP accumulation was performed using ImageJ software (v.1.8.0, NIH, Bethesda, MD, USA). A color-based mask was generated by thresholding the hue-saturation-brightness (HSB) color space, with a fixed saturation threshold of 68 applied consistently across all images to isolate red fluorescent pixels corresponding to PET-NP aggregates. The percentage of the image area occupied by red fluorescent pixels relative to the total section area was calculated as a proxy for PET-NP accumulation. For each experimental group, 10 images were acquired randomly from 3 animals (n = 30 images per group in total). All images were processed under identical acquisition and analysis settings to ensure comparability across groups. Statistical analysis was performed in two steps. First, a two-way ANOVA was applied to assess the effects of dose and time, and their interaction, on PET-NP accumulation. As no significant interaction between the two factors was detected, groups sharing the same dose or the same time point were subsequently compared pairwise using Student’s *t*-test. Differences were considered statistically significant at *p* < 0.05.

For hemocytes exposed ex vivo to PET-NPs, 10 randomly selected microscopic fields were acquired from 5 slides per treatment (5 mg/L; 5 mg/L + anticoagulant; 10 mg/L; 10 mg/L + anticoagulant). The total number of hemocytes and the number of those containing PET-NP were counted manually. The proportion of hemocytes containing PET-NPs was quantified relative to the total number of cells. Differences among treatments were analyzed using two-way ANOVA to assess the effects of dose, anticoagulant, and their interaction (significance set at *α =* 0.01). Additional comparisons among groups were performed using the Student’s *t*-test, with significance set at *α =* 0.05. All statistical analyses were performed using PAST v.2.09 software [49].

## 3. Results

### 3.1. PET-NPs Accumulate in the AK and PK of P. canaliculata After Acute Exposure but Do Not Alter Kidney Morphology

Microscopic analysis of cryostat sections of organs obtained from animals exposed daily to 5 mg/LPET-NP shows no sign of accumulation (Supplementary Figure S5). Injection was therefore selected as the exposure route, as it ensures controlled and reproducible delivery of a defined PET-NP dose directly into the hemolymph. This parenteral approach has been previously employed in our laboratory for exposure to other types of nanoparticles, providing a validated framework for the present investigation [45].

**Figure 1:**
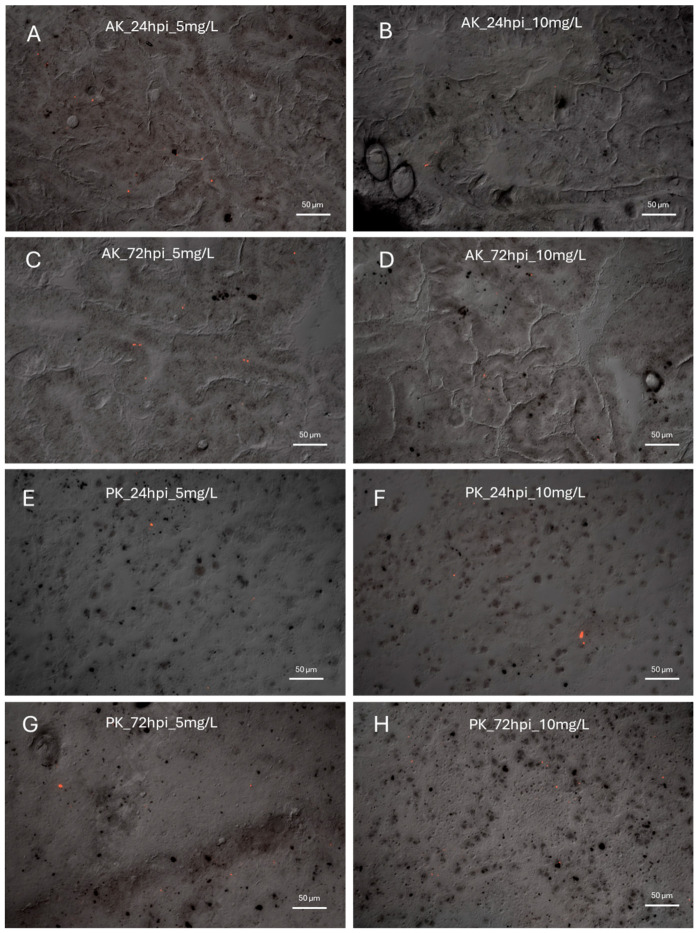
Accumulation of nanoparticles (PET-NPs) in the AK and PK of *P. canaliculata* PET-NPs accumulation was evaluated in AK and PK after 24 hpi (**A**,**B**,**E**,**F**) and 72 hpi (**C**,**D**,**G**,**H**) at concentrations of 5 and 10 mg/L. The presence of PET-NP aggregates within the tissue can be observed PET-NP presence was observed in both AK and PK at all time points and concentrations tested (red dots).

**Figure 2:**
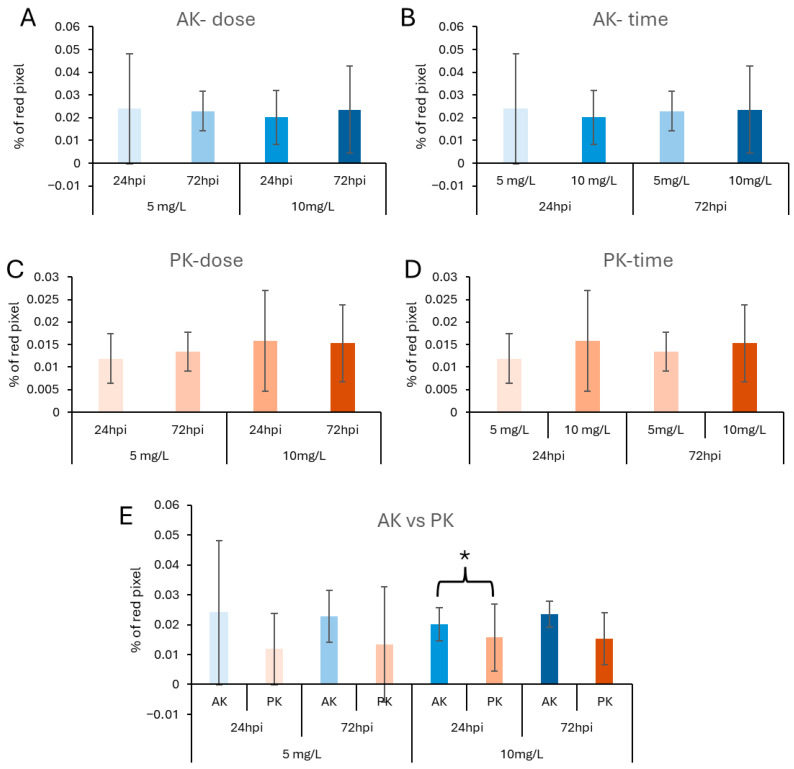
The accumulation of PET-NP aggregates is unaffected by the concentrations used and remains stable over time. PET-NP accumulation in tissues was quantified using image analysis and a mask to calculate the percentage of saturated pixels. In the AK, no significant differences in accumulation were observed with respect to either PET-NPs concentration (**A**) or exposure time (**B**). Similarly, no significant differences were observed in the PK with respect to concentration (**C**) or time (**D**). Direct comparison of the AK and PK under identical exposure conditions revealed significantly lower PET-NP accumulation in the PK at 24 hpi with 10 mg/L PET-NPs (**E**). Groups were considered significantly different at *p* < 0.05 (*).

Microscopic analysis of cryostat sections revealed PET-NP presence in both the AK and the PK at the two tested concentrations (5 and 10 mg/L) and times (24 and 72 hpi) of injected animals (Figure 1). Image analysis performed using a saturation-based mask to quantify the percentage of red pixels (i.e., PET-NP aggregates) per section showed no significant interaction between dose and time on PET-NP accumulation in either organ (*p* > 0.05); moreover, the accumulation of PET-NPs was not dependent on the dose or the time (Figure 2). When PET-NP levels were compared between the two organs under identical exposure conditions, a significantly lower accumulation was observed in the PK after exposure to 10 mg/L PET-NPs for 24 hpi.

**Figure 3:**
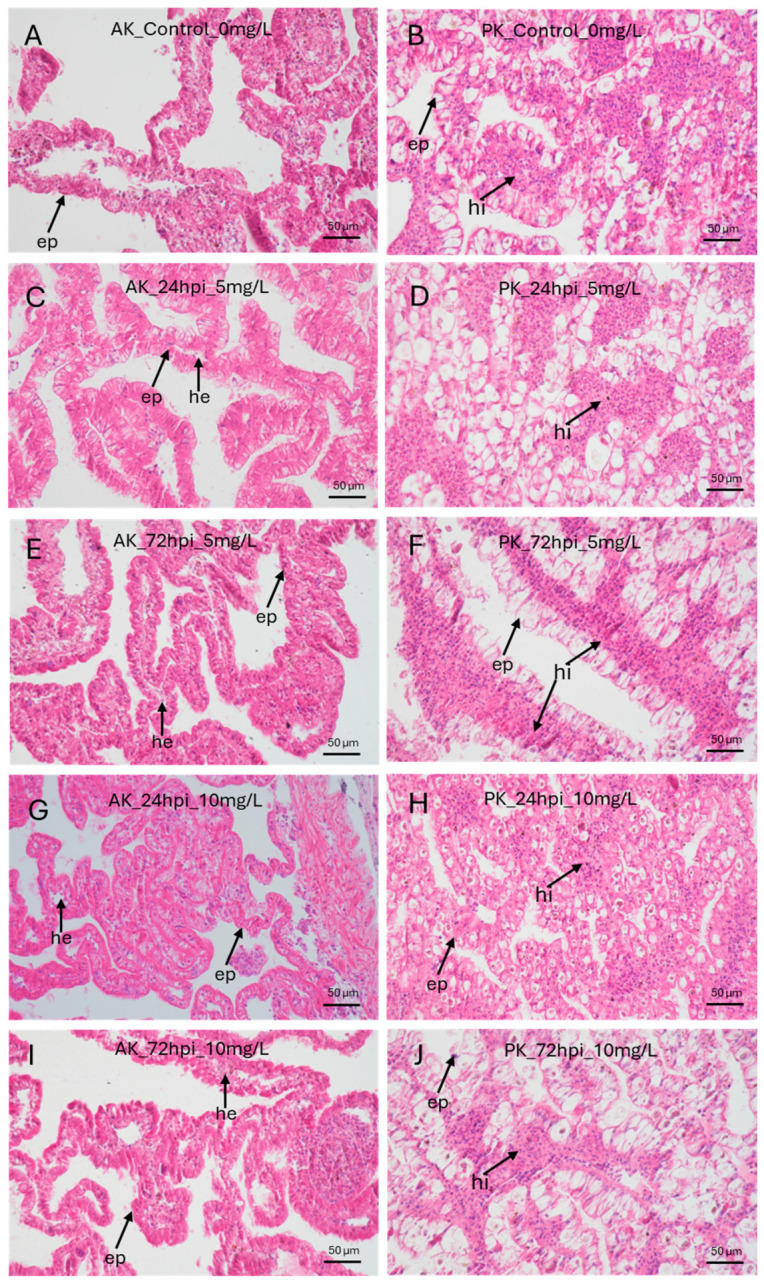
**PET-NPs did not induce histological alterations in the AK or PK.** Hematoxylin and eosin staining was performed on the AK and PK after exposure to 0 mg/L PET-NPs (**A**,**B**), 5 mg/L PET-NPs at 24 h post-injection (hpi; **C**,**D**) and 72 hpi (**E**,**F**), and 10 mg/L PET-NPs at 24 hpi (**G**,**H**) and 72 hpi (**I**,**J**). The AK is made by a ramified parenchyma with a connective core and an external layer of epithelial cells; inside the protrusion, hemocyte-like cells can be found. PK is a tubular parenchymatous organ with a more intricate structure, delimited by a cylindrical epithelium that encloses aggregates of hemocyte-like cells (hemocyte islets). No significant tissue alterations were observed in either the AK or the PK at any exposure time or PET-NP concentration. ep = epithelium; hi = hemocyte islets; he = hemocyte(s).

To gain a better insight into their morphology, portions of treated AK and PK were fixed in Bouin’s solution, dehydrated, and embedded in Paraplast^®^, then stained with hematoxylin and eosin. The histological analysis showed no morphological alterations after PET-NP exposure in either the AK or the PK across all treatments. The epithelia were intact, the tissue stainability was preserved, and the cells presented a regular morphology. In the PK, no alterations were observed in the hemocytic islets (Figure 3).

### 3.2. PET-NPs Do Not Induce Expression of Stress and Inflammation-Related Genes in AK and PK

**Figure 4:**
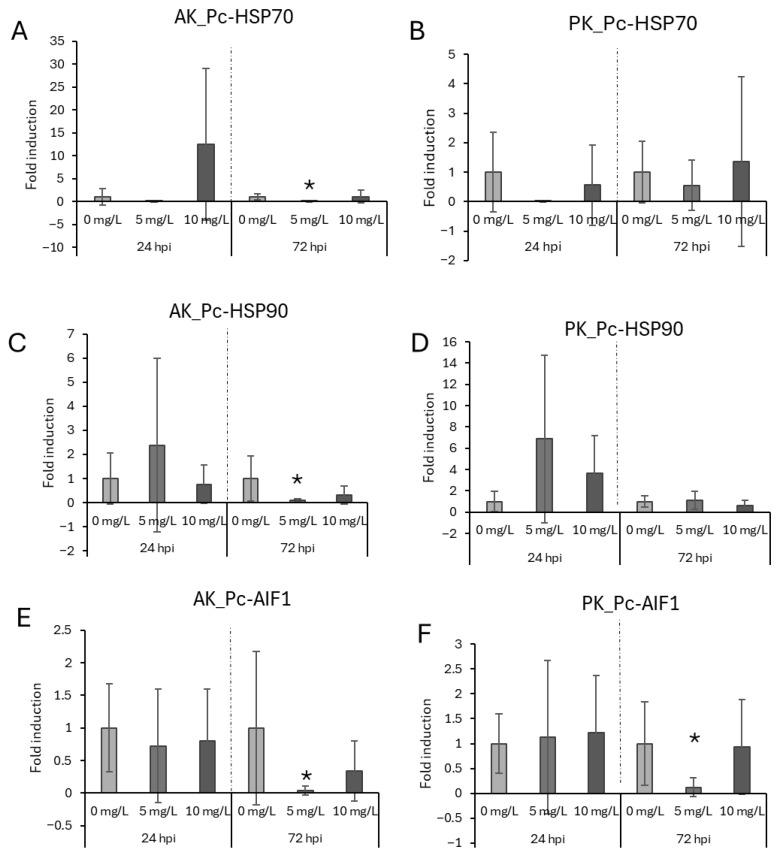
Expression of stress- and inflammation-related genes. Gene expression associated with stress (Pc-HSP70; Pc-HSP90) and inflammation (Pc-AIF1) was analyzed in the AK (**A**,**C**,**E**) and PK (**B**,**D**,**F**) following exposure to 5 mg/L and 10 mg/L PET-NPs at 24 and 72 hpi. In the AK, PC-HSP70 expression was significantly downregulated at 72 hpi after exposure to 5 mg/L PET-NPs (**A**), whereas no significant changes were observed in the PK (**B**). Similarly, Pc-HSP90 expression in the AK was significantly downregulated at 72 hpi following exposure to 5 mg/L PET-NPs (**C**), while no significant modulation was detected in the PK (**D**). Pc-AIF1 expression was significantly downregulated at 24 hpi with 10 mg/L NP exposure in both AK (**E**) and PK (**F**). Groups were considered significantly different at *p* < 0.05 (*).

Gene expression analysis revealed a modulation of stress- and inflammation-related genes in response to PET-NP exposure, with distinct responses observed in AK and PK (Figure 4). In AK, Pc-HSP70 expression was significantly downregulated at 72 hpi following exposure to 5 mg/L PET-NPs (*p* < 0.05). No statistically significant changes in Pc-HSP70 expression were detected in the other experimental conditions. The mean value of Pc-HSP70 expression in AK at 24 hpi after exposure to 10 mg/L PET-NPs was 12.5-fold higher than in controls. However, the high intragroup variability prevented this increase from reaching statistical significance (*p* > 0.05). Similarly to Pc-HSP70, Pc-HSP90 expression was significantly down-regulated in AK at 72 hpi following exposure to 5 mg/L PET-NPs (*p* < 0.05), with no other significant differences observed under the other exposure conditions. In PK, relevant intragroup variability was observed, but neither Pc-HSP70 nor Pc-HSP90 exhibited statistically significant changes under any of the experimental conditions.

In both AK and PK, Pc-AIF1 expression was significantly downregulated at 72 hpi of 5 mg/L PET-NPs, and no other doses or time points changed the expression of the inflammation-related gene (Figure 4).

### 3.3. Circulating Hemocyte of Different Morphologies Phagocytized PET-NPs

**Figure 5:**
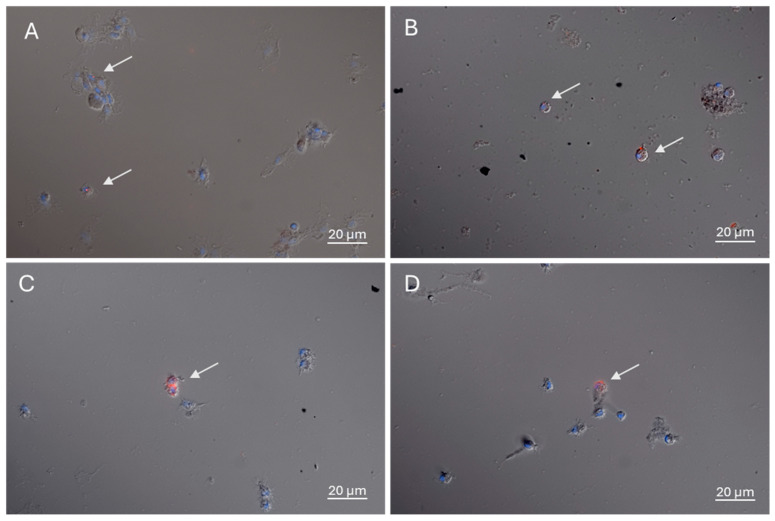
Circulating hemocytes phagocytized PET-NPs. Hemocytes from snails injected with 5 mg/L PET-NPs show phagocytic activity at both 24 and 72 hpi (**A**,**B**). Similarly, the hemocytes from snails injected with 10 mg/L PET-NPs exhibit phagocytosis of PET-NP aggregates at 24 and 72 hpi (**C**,**D**). PET-NP aggregates were visible in red, while nuclei were stained with DAPI (blue).

**Figure 6:**
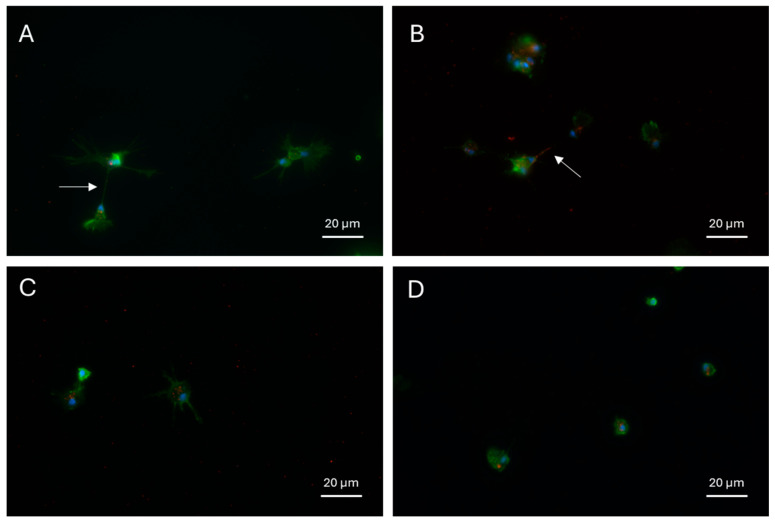
Hemocytes with distinct morphologies phagocytose PET-NPs. The images show circulating hemocytes that have internalized PET-NPs (red). Nuclei are stained with DAPI (blue), and actin is labeled by immunofluorescence (green). PET-NP uptake is observed in well-adherent cells displaying pseudopodia (**A**–**C**), which appear to transfer PET-NPs through cytoplasmic protrusion (white arrow), as well as in smaller, round hemocytes (**D**).

The hemolymph collected at 24 or 72 hpi from snails injected with 5 or 10 mg/L PET-NPs was examined by fluorescence microscopy. Hemocytes phagocytosing PET-NPs were observed at both tested concentrations at 24 and 72 hpi (Figure 5). Immunofluorescence labeling of the cytoskeletal actin was performed to better characterize the morphology of the hemocytes involved in PET-NP phagocytosis. The immunocytochemical analysis revealed that the hemocytes internalizing PET-NPs displayed distinct morphologies (Figure 6). PET-NP uptake was observed both in well-adherent cells displaying pseudopodia, which appeared to hook up to PET-NPs through cytoplasmic protrusion [50], and in smaller, round hemocytes. The analysis of hemocytes from injected animals was performed as a qualitative assessment, aimed at confirming the occurrence of PET-NP internalization in circulating immune cells under in vivo conditions. Quantification of phagocytic activity was therefore carried out exclusively in the ex vivo incubation experiment (Section 3.4), where the use of pooled hemolymph from multiple donors provided more standardized and controlled conditions.

### 3.4. Hemocyte Can Accumulate PET-NPs Ex Vivo in a Dose-Dependent Manner and Treatment with Anticoagulant Reduces This Ability

**Figure 7:**
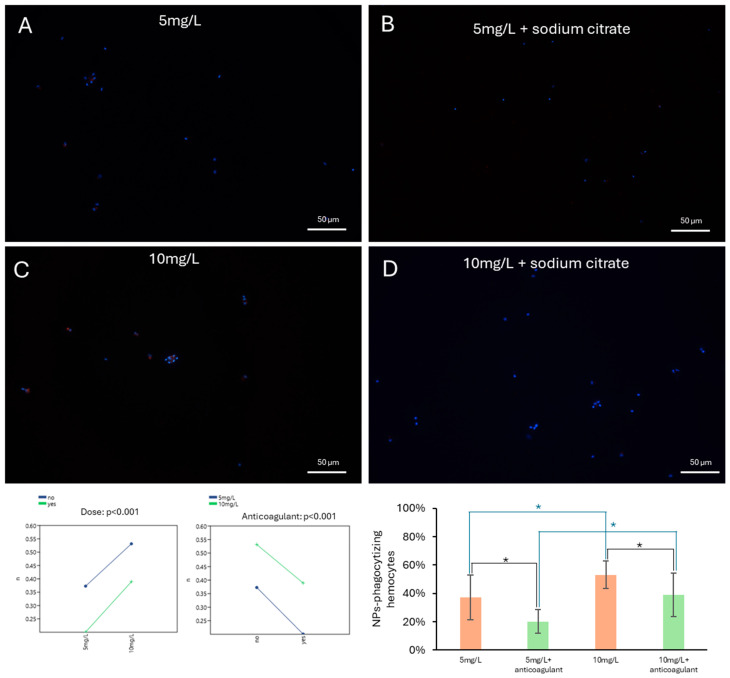
Ex vivo phagocytosis of PET-NPs. Hemocytes exposed ex vivo to 5 mg/L or 10 mg/L of PET-NPs for 30 min, either in the absence (**A**,**C**) or presence (**B**,**D**) of an anticoagulant, showed PET-NP intracellular accumulation. The percentage of hemocytes accumulating PET-NPs was significantly affected by both the PET-NP concentration and the presence of the anticoagulant. Higher PET-NP concentrations resulted in a significantly higher number of phagocytic cells, whereas the presence of sodium citrate had a detrimental effect on phagocytosis. The effects of PET-NP dose and anticoagulant treatment were considered significant at *p* < 0.01 (two-way ANOVA), while differences between individual groups were considered significant at *p* < 0.05 (*) (*t*-test).

Hemocytes collected from control snails were incubated ex vivo for 30 min with 5 or 10 mg/L PET-NPs, in the presence or absence of an anticoagulant (sodium citrate, 10 mM). Under all the experimental conditions, hemocytes were able to phagocytose PET-NPs ex vivo (Figure 7).

Two-way ANOVA revealed a significant effect of PET-NP concentration, with an increased percentage of PET-NP–phagocytosing cells observed at higher doses under ex vivo conditions. There was also a significant effect of anticoagulant treatment (*p* < 0.01), which reduced the phagocytic activity of hemocytes at all the PET-NP concentrations. Exposure to 5 mg/L PET-NPs resulted in 37 ± 16% of cells being phagocytic, a percentage that decreased to 20 ± 8% in the presence of the anticoagulant. Exposure to 10 mg/L PET-NPs resulted in a significantly higher proportion of phagocytic cells (*p* < 0.05), reaching 53 ± 10%. This was reduced to 39 ± 15% by the anticoagulant. Although these results suggest a concentration-dependent trend in hemocyte phagocytic activity under ex vivo conditions and may not fully reflect the complexity of the in vivo immune response.

## 4. Discussion

The pervasive presence of plastic debris in aquatic environments, together with its progressive fragmentation into micro- and nanoscale particles, represents a threat to freshwater ecosystems that warrants further investigation [12,13]. NPs are of increasing concern due to their high bioavailability, ability to cross biological barriers and potential to disrupt cellular and physiological homeostasis, even at low concentrations [17]. Despite growing evidence of NP-induced toxicity across multiple taxa, freshwater systems and invertebrate models remain markedly underrepresented in ecotoxicological research [20,21]. Actually, the focus of environmental quantifications and ecotoxicological studies has been disproportionately on marine systems, meaning the freshwater and wetland environments have been relatively underrepresented, even though they are potentially more contaminated [13,20,51].

Gastropods constitute a large, diverse, and ecologically important group of freshwater invertebrates, which are widely distributed across continents and aquatic habitats [52]. They contribute to nutrient cycling, graze on periphyton, influence energy flow, and serve as prey for higher trophic levels [53]. Several species have been used as sentinel organisms in ecotoxicology studies to evaluate the impact of metals, agrochemicals, pharmaceuticals, and emerging pollutants [54,55]. However, despite their ecological significance and their demonstrated capacity to ingest and bioaccumulate MP/NPs from water, sediment, or food, studies examining the mechanisms of NP toxicity in freshwater gastropods remain surprisingly limited [53] (Rodrigues et al., 2024). Research has shown that gastropod species accumulate MPs/NPs in field and laboratory settings, with particles being found in the respiratory system, foot, visceral mass, intestine, and circulating hemolymph [53]. NPs can be transferred from the digestive tract to hemocytes, potentially affecting immunity, development, or reproduction; transgenerational effects have also been reported, such as NP accumulation in the eggs of *Biomphalaria glabrata* or reduced clutch size in *Lymnaea stagnalis* [56,57]. Nevertheless, despite these initial observations, there are virtually no comprehensive studies investigating the accumulation of NP, their organ-specific effects, immune responses, and the cellular pathways they affect in gastropods.

Against this backdrop, the present study investigated the tissue distribution, cellular effects, and immune responses triggered by acute exposure to PET-NPs in the freshwater gastropod *P. canaliculata* administered by injection. It should be acknowledged that injection bypasses the natural exposure routes of *P. canaliculata*, such as the digestive tract and gills, and may therefore influence the distribution and bioavailability of PET-NPs compared to environmentally realistic scenarios. However, dietary exposure under the conditions tested failed to produce detectable particle accumulation in target organs (Supplementary Figure S5), and immersion exposure was deemed technically unfeasible for the reasons outlined in the Methods. The parenteral route was therefore selected as the most controlled and reproducible approach available for this first investigation.

Our results demonstrate that PET-NPs are rapidly distributed to major excretory and immunological compartments following acute exposure, particularly the anterior (AK) and posterior (PK) kidneys. Cryostat section analyses revealed the presence of PET-NPs in both organs as early as 24 hpi, with persistence up to 72 hpi, regardless of the administered dose. The absence of a clear dose- or time-dependent increase in tissue-associated fluorescence, and thus PET-NP accumulation, might suggest that uptake in these organs may reach saturation rapidly, or that uptake and clearance processes may occur simultaneously, resulting in stable accumulation levels over the investigated timeframe.

Only limited differences were observed between the AK and the PK, with significantly lower accumulation in the PK detected exclusively at the earliest time point. Although no direct measurements of renal physiology, filtration dynamics, or particle transport were performed in the present study, the morphological and functional differences between the two chambers described in the literature for *P. canaliculata* may provide a basis for interpreting this observation. The AK is characterized by regularly arranged transverse folds, intimate contact between blood spaces and epithelium, and an abundance of resident hemocytes, features that have been associated with osmoregulatory functions. The PK, by contrast, has a more capacious lumen, a highly vascularized connective tissue also rich in hemocyte, and is primarily devoted to the elaboration and excretion of nitrogenous waste in the form of uric acid concretions [58].

Reduced early accumulation in the PK at high PET-NP concentrations could indicate a lower retention capacity or more efficient clearance mechanisms in this organ. Despite clear evidence of the presence of PET-NPs in both the AK and the PK, histological analyses did not reveal any detectable morphological alterations following exposure. Thus, under the acute exposure conditions tested here, PET-NPs do not induce evident tissue damage or structural alterations in renal tissues. As to whether these structural and functional differences between PK and AK could contribute to differential PET-NP accumulation, evidence on particle transport and cellular trafficking warrants dedicated investigation in future studies. The absence of histopathological effects could suggest the idea that early or low-level stress responses may occur at a molecular level prior to manifesting as morphological changes. Studies on the effects of MPs/NPs on organ morphology are fewer than studies on gene expression and enzymatic activity, especially for invertebrates. This is also due to the effect of the solvent used in histopathological protocols on MPs/NPs [59]. Furthermore, the histopathological effects of NPs can vary in different studies and species. For example, exposure to NPs in zebrafish larvae induces endoplasmic reticulum stress in the kidneys [60], whereas signs of inflammation and necrosis were found in the kidneys of mice exposed to NPs [61].

In line with this interpretation, gene expression analyses were performed for stress-related genes (Pc-HSP70 and Pc-HSP90) and inflammation-related genes (Pc-AIF1). No positive modulation of these genes was detected in either AK or PK. All of the genes tested were downregulated relative to the controls at 72 hpi with 5 mg/L of PET-NPs. As this expression pattern was observed for all genes within a single experimental condition, it may reflect lower basal expression levels in that group rather than a direct effect of PET-NP exposure. Consistently, the only significant difference detected in the PK concerned Pc-AIF1 expression within the same group. Alternatively, this response may indicate that lower PET-NP doses prompt a delayed regulatory response, potentially linked to adaptive or compensatory mechanisms rather than classical dose-dependent toxicity. The absence of significant transcriptional changes at 10 mg/L, despite high interindividual variability and large mean fluctuations at 24 h post-infection (hpi), may reflect heterogeneous individual responses driven by differences in uptake efficiency, immune status or physiological condition.

Similar non-linear, concentration-dependent transcriptional responses have been reported in other invertebrates. For example, an extensive characterization of HSP70 genes in *Monodonta labio* revealed that three members were strongly upregulated following acute NP exposure at 0.1 mg/L. In contrast, exposure to 10 mg/L resulted in the up- and downregulation of distinct genes [62]. Similarly, NP exposure downregulated an HSP70 gene in the digestive gland of *M. galloprovincialis*, leading to reduced metabolic activity and impaired digestion [32]. Exposure to PET-NPs *in D. melanogaster* larvae caused significant downregulation of HSP70 [19], supporting the hypothesis that NP-induced stress responses are not necessarily linear and may vary depending on concentration, exposure scenario and species-specific sensitivity.

The detection of PET-NPs within the open circulatory system, particularly in AK and PK, suggests that this organism is capable of accumulating particles in organs associated with immune and/or phagocytic functions. The posterior kidney is known to sequester exogenous agents within specialized structures called hemocyte islets, which are composed of aggregates of phagocytic cells involved in non-self recognition and elimination [63]. Technical limitations prevented PET-NP localization within hemocyte islets in the present study. Paraffin embedding requires tissue clearing with organic solvents such as xylene, which can weaken, swell, or dissolve plastics. This can lead to NP loss during histological processing, despite good preservation of tissue morphology. Consequently, to overcome this limitation, we investigated the phagocytic capacity of circulating hemocytes.

Analysis of circulating hemocytes provided key insights into the organism’s response to PET-NP exposure. Hemocytes capable of internalizing PET-NPs were detected in injected animals at both concentrations (5 and 10 mg/L) and time points (24 and 72 hpi), indicating that phagocytosis is a rapid and persistent route of PET-NP internalization. This is supported by the documented role of invertebrate hemocytes in NP phagocytosis [36,64,65]. In *M. galloprovincialis,* for instance, hemocytes internalize NPs via endocytic and non-endocytic pathways, primarily accumulating them within lysosomes [36].

Immunofluorescence analyses revealed that hemocytes with distinct morphologies, including well-adherent cells with pseudopodia and smaller, rounded cells, internalize PET-NPs. This morphological diversity suggests that multiple hemocyte subtypes are involved in NPs uptake and transport, indicating a complex immune response rather than a single specialized phagocytic population. Similar results have been reported in *Artemia salina*, where amino-modified polystyrene NPs were primarily phagocytosed by plasmatocytes and granulocytes at a rate of 22.64% [64]. In *Perna viridis*, both granular and agranular hemocytes efficiently internalize NPs, with granulocytes preferentially using clathrin-mediated pathways and agranulocytes using caveolin-mediated pathways, depending on particle size [65]. Furthermore, NPs uptake has been demonstrated to impact the composition of hemocyte subpopulations, promoting the differentiation of agranulocytes into granulocytes [65].

Furthermore, imaging with an actin label revealed apparent intercellular transfer of PET-NPs via cytoplasmic protrusions. This finding aligns with recent research on *P. viridis*, which suggests that NPs internalized by granulocytes are redistributed among hemocytes via size-dependent transfer pathways, including extracellular vesicles and tunneling nanotubes [50]. In the literature, this process has been linked to the transfer of mitochondria to stressed cells, which contributes to maintaining the redox balance, enhancing phagocytic activity, and modulating the immune response [50].

Ex vivo exposure experiments further confirmed the capacity of hemocytes to phagocytose PET-NPs, with a higher proportion of phagocytic cells observed at the higher tested concentration. Although ex vivo conditions do not fully recapitulate the complexity of the in vivo hemolymph environment, the incubation of isolated molluscan hemocytes represents a well-validated approach in invertebrate immunology, extensively used to characterize phagocytic responses and interactions with foreign particles under controlled conditions [66,67,68,69]. This approach allowed precise control of PET-NP concentration and exposure duration, and provided a standardized framework for quantitative comparisons that would not be feasible from hemolymph sampled directly from individual animals.

A significant reduction in the proportion of phagocytic cells was observed in the presence of an anticoagulant, highlighting the importance of hemocyte adhesion and coagulation-related processes in PET-NP uptake. The fact that anticoagulant treatment measurably reduced phagocytic activity suggests that NP internalization relies on active cellular processes that can be disrupted by interference with hemolymph coagulation dynamics, rather than occurring through passive diffusion alone. While anticoagulants are routinely employed in hemolymph collection protocols to prevent clotting, their potential impact on hemocyte functional activity has rarely been explicitly evaluated in gastropods. The significant effect observed here highlights the importance of considering this factor when designing and interpreting ex vivo phagocytosis assays in *P. canaliculata*. These findings provide a methodological foundation for future co-incubation experiments, such as simultaneous exposure of hemocytes to NPs and fluorescently labeled bacteria, which will allow direct investigation of how NP exposure modulates innate immune recognition and antimicrobial responses in this species.

Overall, our findings suggest that exposure to PET-NPs results in the rapid uptake and accumulation of the particles in renal tissues without causing overt tissue damage. This process simultaneously induces measurable molecular and cellular responses that are organ-specific, time-dependent and non-linear with respect to dose. Integrated analysis of tissue accumulation, gene expression and hemocyte behavior suggests that the immune system could have a role in managing PET-NPs despite the inert nature of the administered material. Hemocytes appear to be possible contributors of PET-NP transport and sequestration within target organs.

The relatively low impact observed on the health of the model organism, *P. canaliculata*, seems to be consistent with its high physiological plasticity and tolerance of environmental stressors, as well as its robust stress responses [25]. This species can thrive in heavily contaminated waters and accumulate high concentrations of heavy metals in its gills with minimal mortality. It can also exhibit histopathological alterations in its digestive tract without compromising survival [26]. Similarly, the limited impact of NPs on egg development, shell growth and hatching rates has been reported in other freshwater gastropods, such as *Lymnaea stagnalis*, even under prolonged exposure scenarios [57]. However, whether the observed low toxicity genuinely reflects the intrinsic tolerance of *P. canaliculata* or is attributable to the specific experimental conditions tested remains to be determined, and future studies employing longer exposure times and broader concentration ranges will be needed to clarify this aspect.

Future studies addressing longer exposure periods and chronic conditions are essential in order to determine whether these early responses represent effective adaptive mechanisms or the onset of long-term dysregulation.

## 5. Conclusions

In conclusion, this study sheds light on the fate of PET-NPs in a freshwater gastropod model of *P. canaliculata* for the first time. It demonstrates that acute exposure results in the rapid uptake and accumulation of PET-NPs in renal tissues, without causing obvious histopathological damage. Integrating tissue distribution, expression profile, and cellular-level suggests a possible role of the immune system, particularly circulating hemocytes, in the scavenging, transport, and sequestration of NPs. The absence of clear dose-dependent toxicity, coupled with the observed non-linear and time-dependent molecular responses, suggests the activation of early regulatory or adaptive mechanisms rather than classical toxic effects. The low overall impact on organismal health seems to be consistent with the high physiological plasticity and stress tolerance of *P. canaliculata*, thereby strengthening its suitability as a resilient yet informative model for NPs research in freshwater systems. These findings underline the importance of incorporating immune-mediated processes into ecotoxicological evaluations of NPs, and highlight the necessity of future studies addressing chronic exposure scenarios to determine whether early responses lead to long-term adaptation or delayed dysfunction.

Nevertheless, some limitations of the present study should be acknowledged. First, the parenteral route of administration, while ensuring precise and reproducible dosing, does not replicate the environmental exposure routes most relevant to wild populations, such as dietary uptake or waterborne exposure. Second, the acute nature of the experiment does not allow conclusions to be drawn regarding the effects of chronic or repeated exposure, which are more ecologically relevant given the persistent nature of plastic contamination in freshwater environments. Future studies employing environmentally realistic exposure routes and chronic exposure scenarios will be essential to fully characterize the ecotoxicological impact of PET-NPs on *P. canaliculata* and freshwater gastropods more broadly.

## Figures and Tables

**Table 1:** Table of primers for RT-qPCR reactions.

Gene	Primer Sequence
*Pc*RpL5	F: 5′-CGTATGCCAGAATTGAGGGT-3′ R: 5′-CAACATCCAAGTATGCACGG-3′
*Pc*HPS70	F: 5′-ATGACGCTCTTCACTGGCTAGA-3′ R: 5′-ACTTCTTCTACAGTTGGTCCTCCA-3′
*Pc*HSP90	F: 5′-TTCAAGCTGGGGCCGACATT-3′ R: 5′-AGTGCCACGTCCCAATTTGC-3′
*PcAIF1*	F: 5′-GCTACTGGCGCAAAGCCTAA-3′ R: 5′-TTGTGGCAGTTCCTCATCAC-3′

## Data Availability

The original contributions presented in this study are included in the article/Supplementary Materials. Further inquiries can be directed to the corresponding author.

## Supplementary Materials

The following supporting information can be downloaded at: https://www.mdpi.com/article/10.3390/jox16030088/s1, Figure S1: Accumulation of nanoparticles (PET-NPs) in the AK and PK of *P. canaliculate,* original image from Figure 1; Figure S2: PET-NPs did not induce histological alterations in the AK or PK. Original images of Figure 3; Figure S3: Circulating hemocytes phagocytized PET-NPs. Original images of Figure 5; Figure S4: Hemocytes with distinct morphologies phagocytose PET-NPs. Original images of Figure 6; Figure S5: Ex vivo phagocytosis of PET-NPs. Hemocytes exposed ex vivo to 5 mg/L or 10 mg/L of PET-NPs for 30 min, either in absence or presence of an anticoagulant, showed PET-NP intracellular accumulation. Original images of Figure 7A-D; Figure S6: Absence of nanoparticles (PET-NPs) in the Anterior Kidney, Posterior Kidney, Digestive Gland (DG) and Stomach (ST) of *P. canaliculata*; Figure S7: Absence of nanoparticles (PET-NPs) in the Anterior Kidney, Posterior Kidney, Digestive Gland (DG) and Stomach (ST) of *P. canaliculata*. Original images of Figure S6.
