# Supplementary material for: Renal Accumulation and Hemocyte-Mediated Internalization After Acute Exposure to Injected Polyethylene Terephthalate Nanoplastics (PET-NPs) in the Freshwater Gastropod Pomacea canaliculata
Source: J Xenobiot. 2026 May 19;16(3):88. doi: 10.3390/jox16030088 (PMC13214758; doi:10.3390/jox16030088)

Journal of *Xenobiotics*

MDPI

Supplementary Materials: Renal Accumulation and Hemocyte-Mediated Internalization After Acute Exposure to Injected Polyethylene Terephthalate Nanoplastics (PET-NPs) in the Freshwater Gastropod Pomacea canaliculata

Anita Ferri, Sandro Sacchi, Chiara Losi, Martina Amico, Nicola Franchi, Davide Malagoli

Figure S1: Accumulation of nanoparticles (PET-NPs) in the AK and PK of *P. canaliculate,* original image from figure 1.

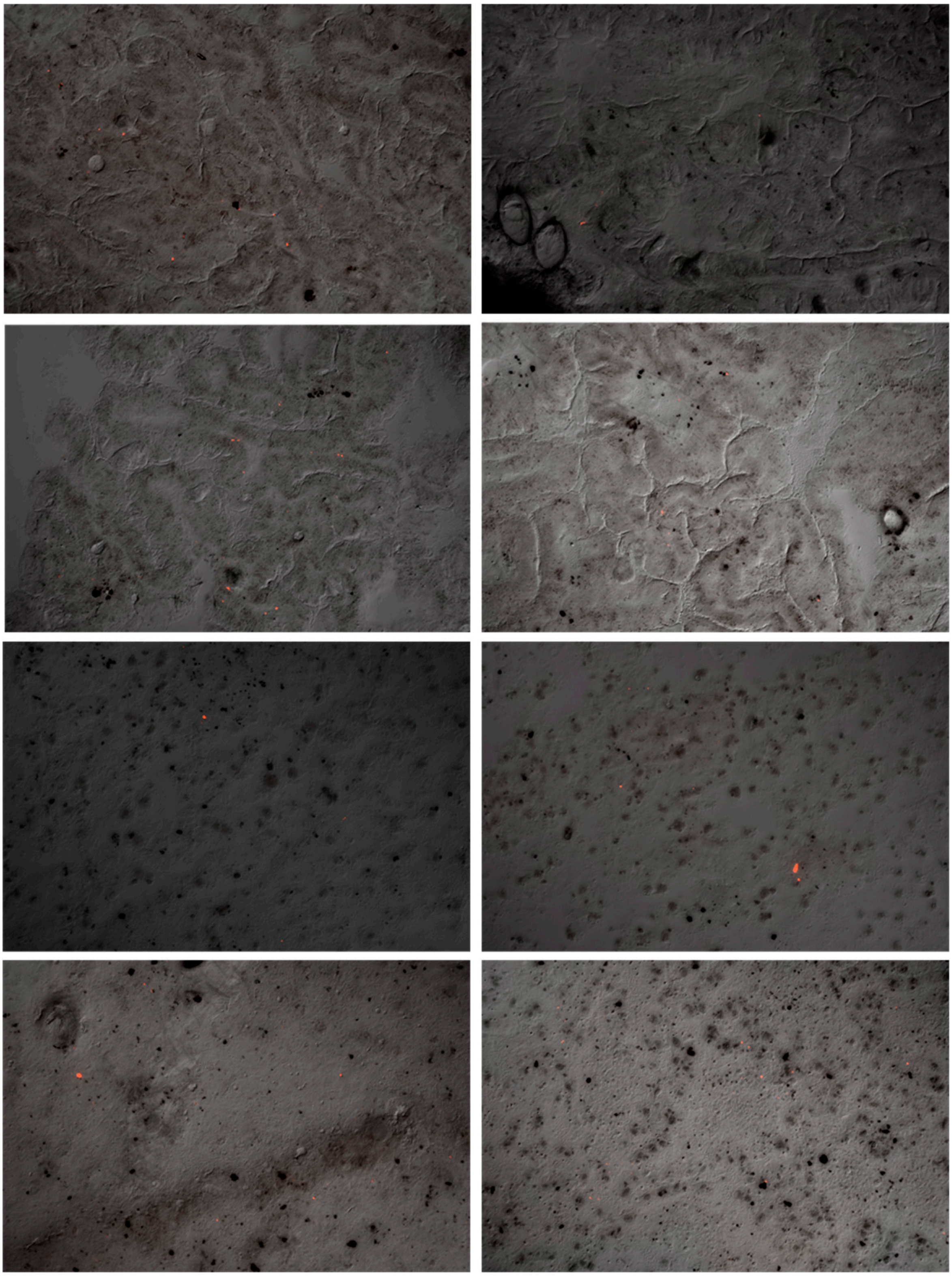

Figure S2: PET-NPs did not induce histological alterations in the AK or PK. Original images of figure 3.

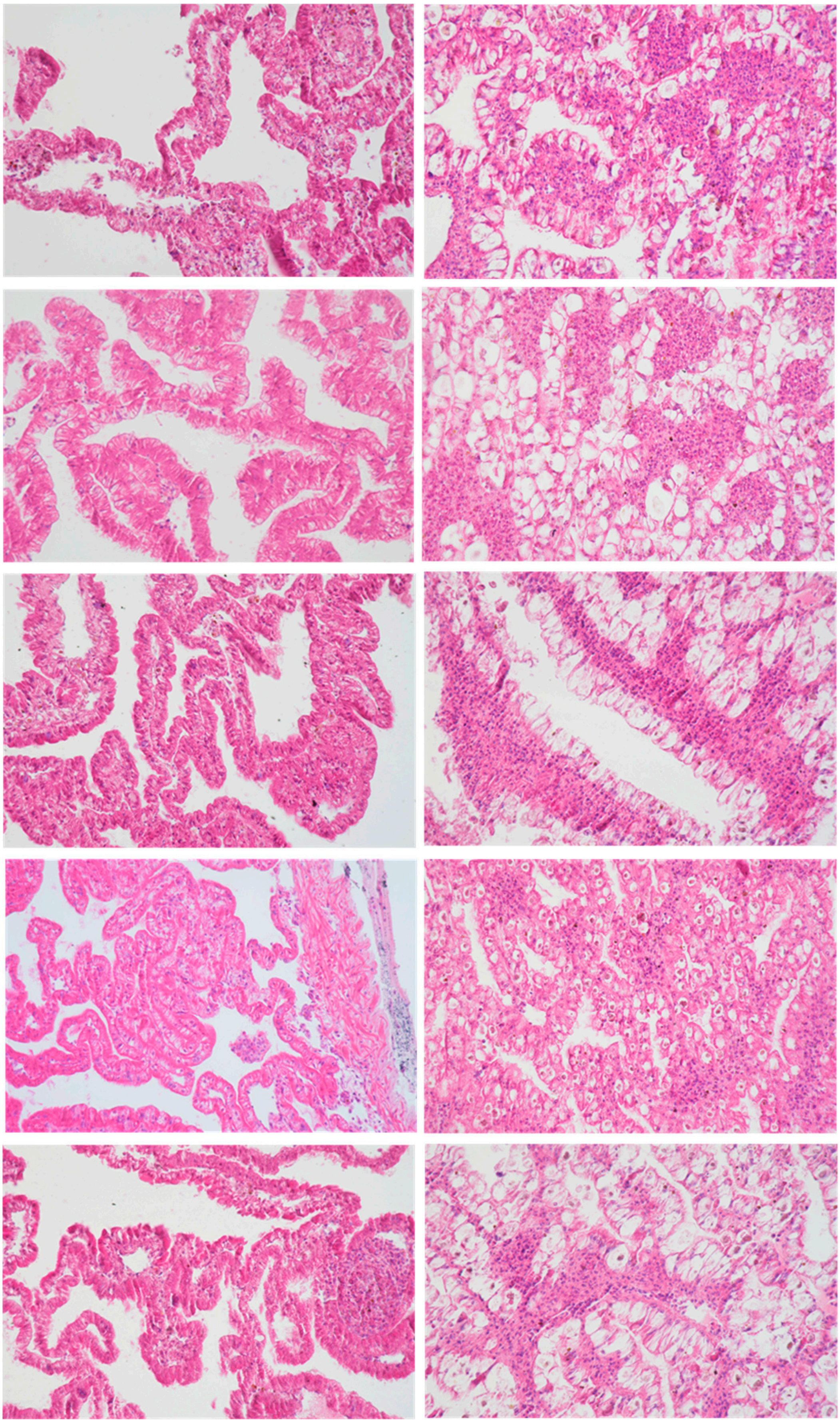

Figure S3: Circulating hemocytes phagocytized PET-NPs. Original images of figure 5.

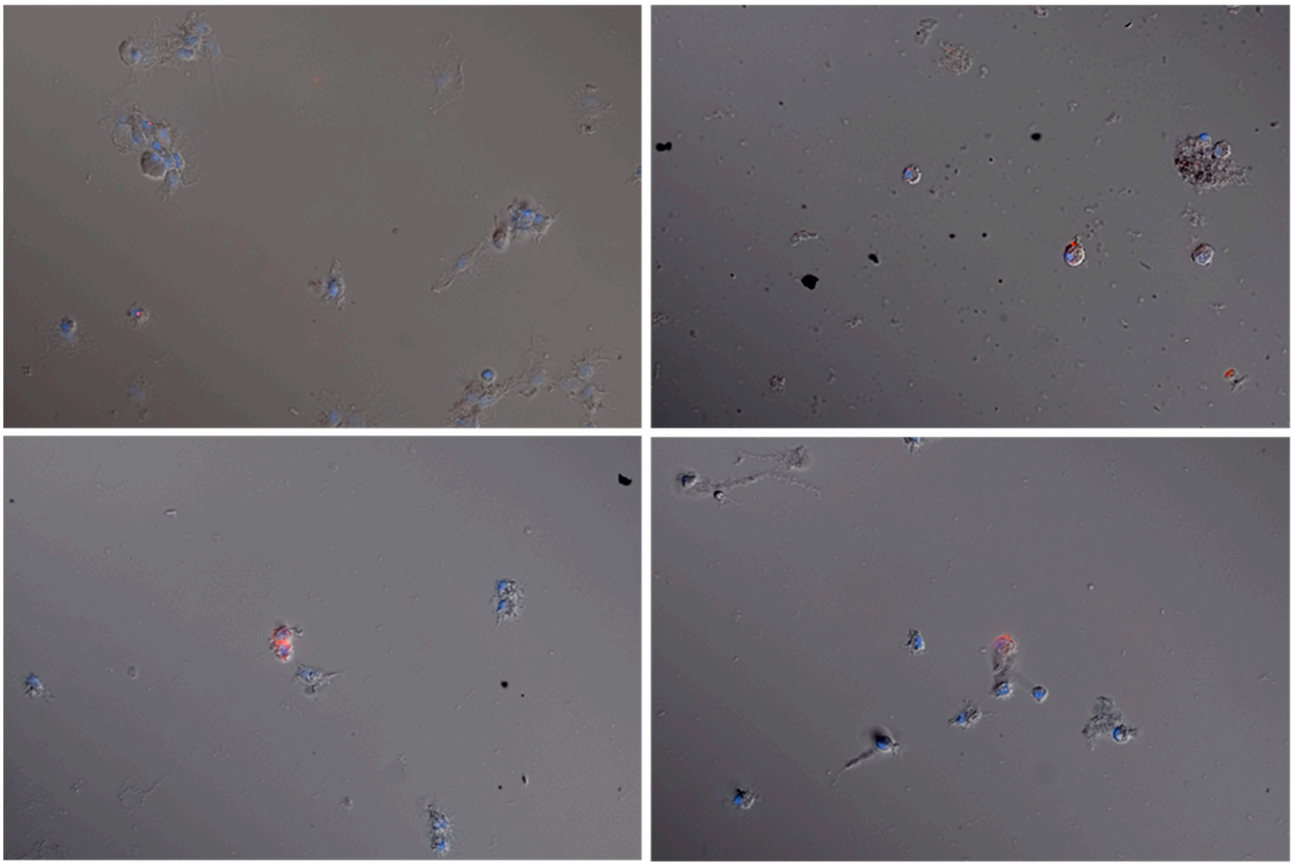

Figure S4: Hemocytes with distinct morphologies phagocytose PET-NPs. Original images of figure 6.

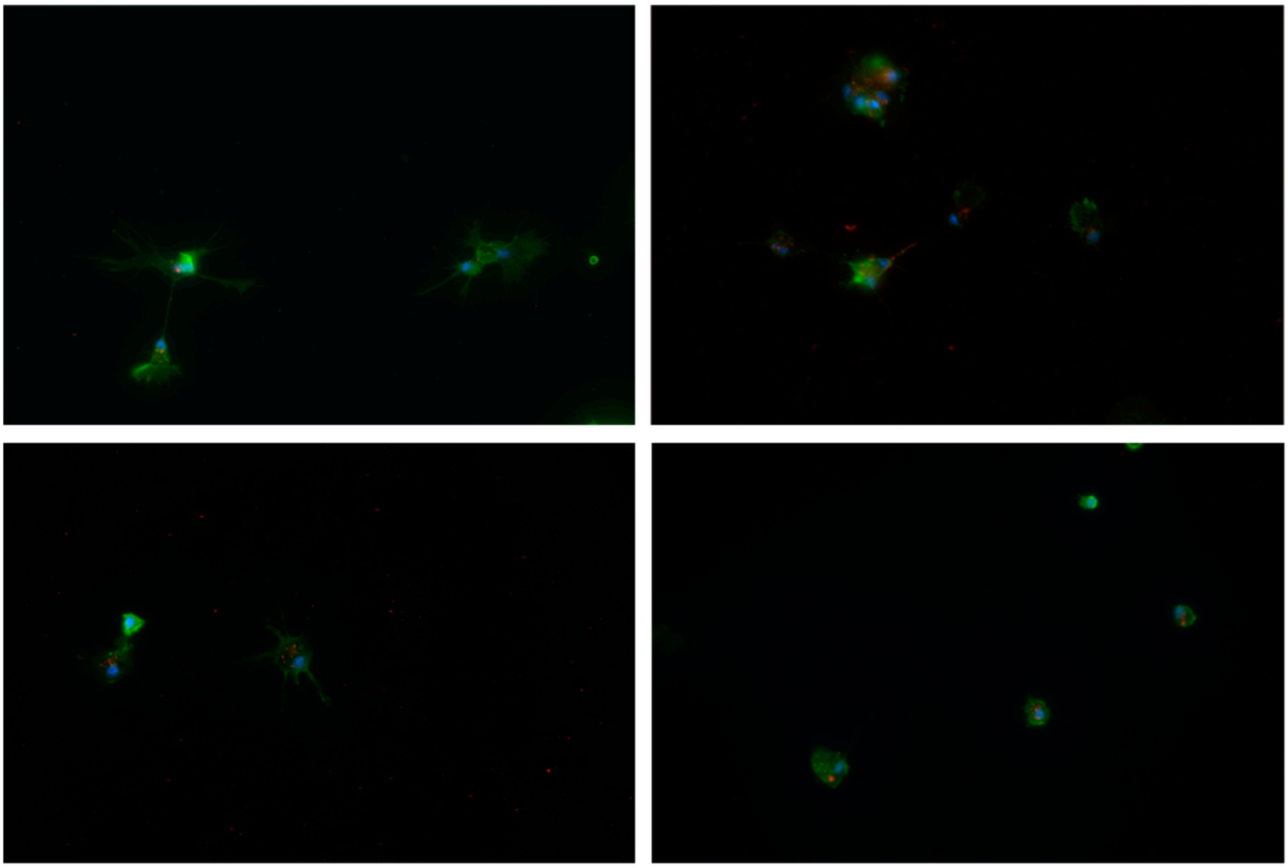

Figure S5: Ex vivo phagocytosis of PET-NPs. Hemocytes exposed ex vivo to 5 mg/L or 10 mg/L of PET-NPs for 30 min, either in absence or presence of an anticoagulant, showed PET-NP intracellular accumulation.. Original images of figure 7A-D.

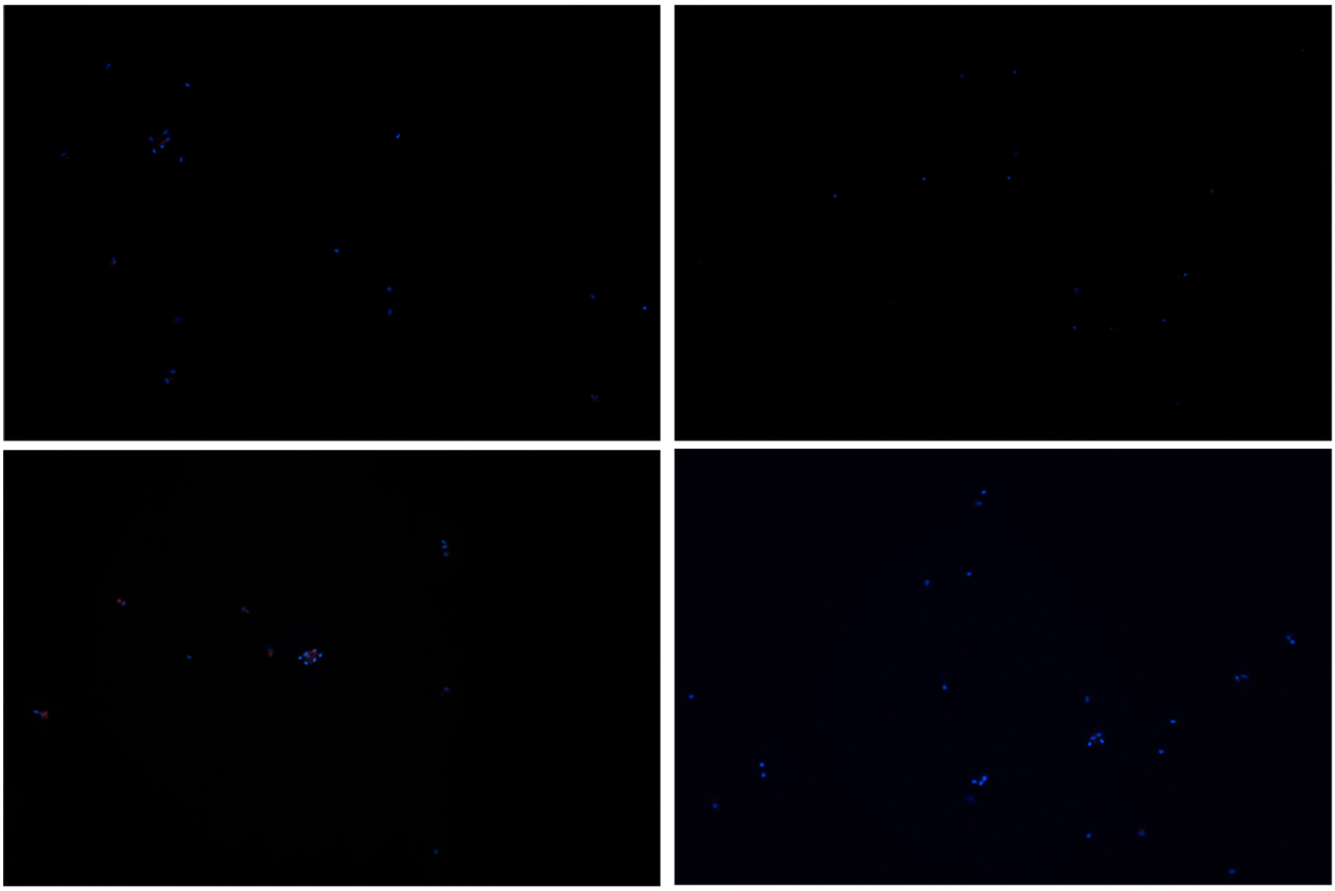

Figure S6: **Absence of nanoparticles (PET-NPs) in the Anterior Kidney, Posterior Kidney, Digestive Gland (DG) and Stomach (ST) of *P. canaliculata*** PET-NPs accumulation was evaluated in AK (A), PK (B), DG (C) and ST (D) after 1 week exposure to 5mg/L by feeding. No presence of PET-NP aggregates within the tissue has been observed

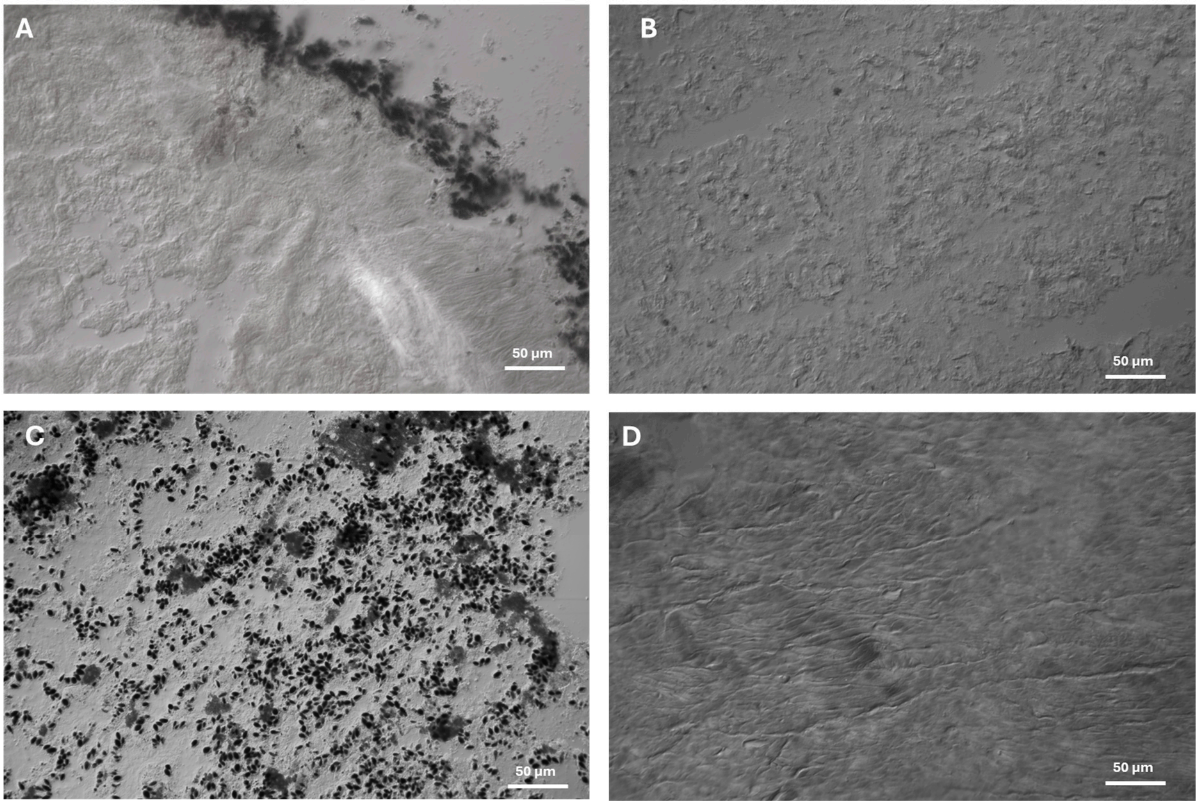

Figure S7: **Absence of nanoparticles (PET-NPs) in the Anterior Kidney, Posterior Kidney, Digestive Gland (DG) and Stomach (ST) of *P. canaliculata***. Original images of figure S6

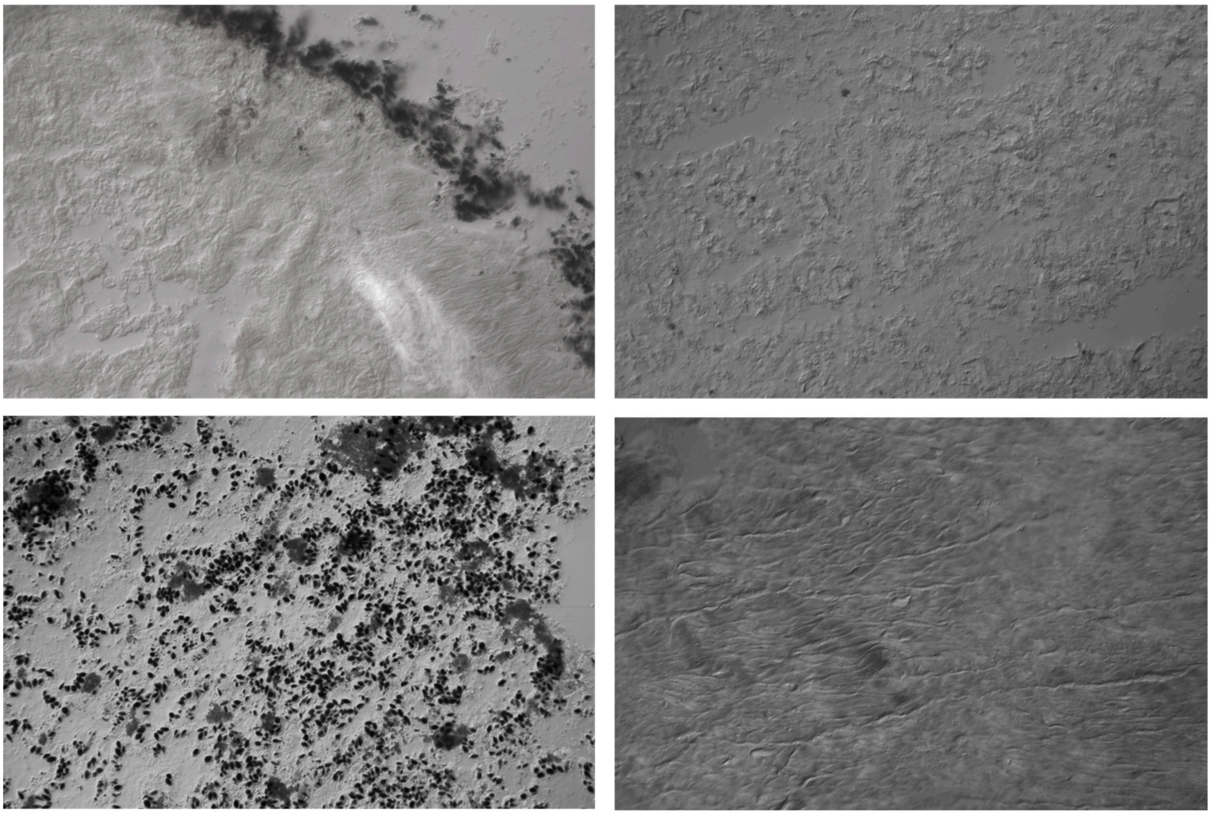

Supplement: Supplementary file 1 [file jox-16-00088-s001.zip › jox-4200030-supplementary.pdf]
